# A Comparison of Online and Blended Learning for Improving Trauma-Informed Practice Education Outcomes

**DOI:** 10.1192/bjo.2025.10295

**Published:** 2025-06-20

**Authors:** Magd Nojoum

**Affiliations:** Central North West London, London, United Kingdom

## Abstract

**Aims:** This study aimed to compare the effectiveness of online and blended learning in improving doctors’ understanding, recognition of trauma signs and symptoms, and confidence in applying trauma-informed practice. The goal was to determine which teaching method leads to greater self-reported improvements.

**Methods:** The online teaching method was delivered through Microsoft Teams. The blended learning programme consisted of both an online component via MS Teams and in-person attendees. Both methods encouraged discussion and interaction. Sessions were organised during established academic time slots for resident doctors. Pre- and post-teaching questionnaires, using a Likert scale, were administered to doctors with varying levels of experience, working across different roles. Results were analysed by calculating the percentage of participants’ agreement in relation to key statements examining understanding, confidence, awareness, and recognition of signs and symptoms of trauma response, before and after the teaching intervention. Differences in sample size and participants’ experience were considered when interpreting the results.

**Results:** Blended learning showed significantly greater improvements compared with online learning. In the blended learning group, the percentage of participants who strongly agreed that their understanding had improved rose from 0% to 60%, while the online learning group increased from 0% to 20%. For recognizing trauma signs and symptoms, the blended learning group showed an increase from 14% to 100%, compared with an increase from 0% to 20% in the online group. Confidence also improved more in the blended learning group, rising from 0% to 40%, compared with an increase from 0% to 20% in the online group.

**Conclusion:** These findings suggest that blended learning is a more effective teaching method for improving understanding, recognition, and confidence in trauma-informed practice education outcomes compared with online learning. However, the variation in participants’ professional backgrounds and experience likely influenced the results, with more experienced doctors potentially benefiting more from certain aspects of the teaching. Future efforts should focus on tailoring blended learning approaches to participants’ experience levels and expanding sample sizes to confirm these findings.

